# Skepticism towards advancing VR technology – student acceptance of VR as a teaching and assessment tool in medicine

**DOI:** 10.3205/zma001496

**Published:** 2021-09-15

**Authors:** Steffen Walter, Robert Speidel, Alexander Hann, Janine Leitner, Lucia Jerg-Bretzke, Peter Kropp, Jakob Garbe, Florian Ebner

**Affiliations:** 1 Ulm University Hospital, Department of Psychosomatic Medicine and Psychotherapy, Medical Psychology Section, Ulm, Germany; 2 University of Ulm, Faculty of Medicine, Competence Center eEducation in Medicine, Ulm, Germany; 3 University Hospital Würzburg, Medical Clinic and Policlinic II, Gastroenterology, Würzburg, Germany; 4 Rostock University Medical Center, Institute for Medical Psychology and Medical Sociology, Rostock, Germany; 5 Halle University Hospital, Department of Internal Medicine I, Halle, Germany; 6 Helios Amper Hospital Dachau, Dachau, Germany

**Keywords:** virtual reality, virtual patient, medical training, future, acceptance

## Abstract

**Objective:** The high didactic potential of Virtual Reality (VR) contrasts with the point of view of students that the technology only has a relatively low significance for current and future teaching. This discrepancy was studied in a differentiated manner in order to gear the further development and implementation of VR towards the target group.

**Methods: **From January 2020 to July 2020, medical students (*N=318*) were asked to watch ten videos online and rate them on the basis of acceptance indicators (e.g., fun and fairness). Using obstetrics as an example, the videos demonstrated five levels of VR technology functionality (e.g., haptic and adaptive feedback), some of which were visionary, in two use scenarios (teaching and the OSCE). The individual and aggregate indicators were compared with non-parametric testing procedures across application scenarios, functional levels and genders. In addition, correlations between the acceptance and the factors of semester, age, computer affinity, and previous VR experience were analyzed.

**Results: **Across all functional levels, VR was more likely to be accepted in the classroom than in the OSCE. Comparisons across functional levels also revealed that the VR ready to be marketed was significantly more accepted than the visionary functions. This skepticism toward advancing VR technology was most pronounced with regard to the vision of autonomous VR examinations and among female students with a low computer affinity.

**Conclusion:** The results suggest that the students’ reservations are due to a lack of experience with the VR technology. In order for young physicians to become familiar with the technology and to be able to use it competently in the everyday clinical practice in the future, VR should not only be used as a teaching tool but also be part of the curriculum. Practical examinations using VR, on the other hand, are only recommended once the technology has become established in teaching and has been proven to be reliable.

## Zusammenfassung

**Zielsetzung: **Das hohe didaktische Potenzial von VR steht im Kontrast zur studentischen Einschätzung, dass die Technologie nur eine relativ geringe Bedeutung für die gegenwärtige und zukünftige Lehre hat. Diese Diskrepanz wurde differenziert untersucht, um die Weiterentwicklung und Implementierung von VR zielgruppenorientiert auszurichten.

**Methodik:** Von Januar bis Juli 2020 wurden Medizinstudierende (N=318) gebeten, zehn Videos online anzusehen und anhand von Akzeptanzindikatoren (z.B. Spaß und Fairness) zu bewerten. Am Beispiel der Geburtshilfe demonstrierten die Videos fünf teils visionäre Funktionsgrade der VR-Technologie (z.B. haptisches und adaptives Feedback) in zwei Einsatzszenarien (Lehre und OSCE-Prüfung). Die einzelnen und aggregierten Indikatoren wurden mit nicht-parametrischen Testverfahren zwischen den Einsatzszenarien, Funktionsgraden und Geschlechtern verglichen. Darüber hinaus wurden Zusammenhänge zwischen der Akzeptanz und den Faktoren Fachsemester, Alter, Computeraffinität und VR-Vorerfahrung analysiert.

**Ergebnisse:** Über alle Funktionsgrade hinweg wurde VR in der Lehre eher akzeptiert als in der OSCE-Prüfung. Die Vergleiche zwischen den Funktionsgraden offenbarten außerdem, dass der marktreife Status Quo von VR signifikant besser angenommen wurde als die visionären Funktionen. Diese Skepsis gegenüber der fortschreitenden VR-Technologie war bei der Vision autonomer VR-Prüfungen und unter weiblichen Studierenden mit geringer Computeraffinität am stärksten ausgeprägt.

**Schlussfolgerung:** Die Ergebnisse legen nahe, dass die Vorbehalte der Studierenden auf mangelnde Erfahrungswerte mit der VR-Technologie zurückzuführen sind. Damit die angehenden Ärzte die Technologie kennenlernen und zukünftig kompetent im Klinikalltag anwenden können, sollte VR nicht nur als Lehrmedium sondern auch als Lerngegenstand curricular eingeführt werden. Praktische Prüfungen in VR empfehlen sich dagegen erst, wenn sich die Technologie in der Lehre etabliert und als verlässlich erwiesen hat.

## 1. The didactic potential of virtual reality

Inert knowledge is not only useless in medicine but life-threatening. Physicians must master specialized knowledge and automate skills in order to make the correct diagnosis and respond adequately in stressful situations. To ensure this level of competency, the National Competency-Based Learning Objective Catalog of Medicine (Nationale Kompetenzbasierte Lernzielkatalog Medizin, NKLM) was introduced in 2015 [http://www.nklm.de]. Students can only achieve the practice-oriented graduate profile of the NLKM if they have sufficient opportunity to practice by using authentic problems from everyday clinical practice during their training [1]. For organizational, financial, and ethical reasons, practicing on patients is insufficient in this regard [2]. One in four medical students admits, for example, to at least one error during training that potentially endangered a patient’s health [3].

Therefore, simulations are used in medical training with which, for example, clinical decision-making and sensorimotor skills can be trained safely. Simulations are understood as learning environments in which students can influence the course of action of scenarios that are as authentic as possible [4], [5]. The most commonly used simulation method in medicine is role-playing with actor-patients [6] or simulation mannequins. However, since these require space, time, and personnel [7], computer-based simulations are becoming increasingly popular as well since they can be used flexibly and allow for an automatic performance assessment [2]. The subject is usually a “virtual patient” who is presented in an interactive video [8] or animation [9], [10]. Depending on the simulation, students can ask the virtual patients about their medical history, physically examine, or treat them. Since the students are usually seated in front of a conventional screen during the exercise and are limited in the actions they can take, these simulations have a relatively high degree of abstraction.

One way to remedy this deficit is the modern virtual reality (VR) simulation. Using VR goggles, students are immersed in a computer-generated world that gives them the impression of being physically present in the learning environment. This immersion, created in part by the high freedom of movement and interaction [11], makes the individual experience and behavior in VR simulations similar to that in a real situation [12]. This opens up the possibility of experiential learning in VR [13], [14] and thus a lasting learning effect. Although the quality of previous research may raise some issues (including small sample size, lack of control groups, and incomplete study reports), quantitative [15], [16] and qualitative [17] meta-analyses largely attest to the didactic added value of VR. It was found with regard to medical education and training, for example, that learning in virtual reality can be more effective than other digital (e.g., videos and online courses) and traditional (e.g., books and lectures) teaching formats [16].

In view of the competence orientation demanded by the NKLM, VR is of great interest in medicine. Every fourth study in the research field of VR has a medical reference [18]. Its use in medical didactics is increasing as well. At the University of Ulm, for example, students have access to a medical VR lab in which they can diagnose and treat virtual patients. In Germany such opportunities are still the exception [19], [20], whereas in English-speaking countries, VR is more widespread and, in some cases, already an integral part of the curriculum [21]. However, the best technology available today does not fully exploit the potential of this technology. If the technical development continues to advance, the currently predominantly audiovisual stimulation in VR [22] will most likely be supplemented with haptic feedback [23], [24]. This will make it possible to not just practice cognitive skills (e.g., process flows in the operating room) but also sensorimotor skills (e.g., physical examinations). Furthermore, the use of artificial intelligence will, in all likelihood, allow for adaptive performance feedback [25], [26] as well as natural verbal communication with virtual patients [9], [10]. The latter will make it possible to practice social skills (e.g., conversational skills with patients) alone in VR alone. The references cited are pilot projects but already paint a vision of a future medical education in which holistic learning experiences in VR are an integral part of the curriculum.

Whether and in what form this vision can become a reality depends in part on how students accept the technology and its future functions. In recent surveys conducted at German universities, students ascribe only a low to moderate importance to VR for both current and future teaching [19], [27]. The objective of this study is to obtain a more differentiated assessment by medical students in order to explore the discrepancy between the didactic potential and the subjectively perceived importance. The findings may help gear the further technical development and integration of VR into the curriculum toward the target group. The following questions were used to conduct the study and structure the presentation of the results:

1. Does the acceptance of VR differ between the teaching and the OSCE application scenarios?
2. Does the acceptance of VR differ between different levels of functionality, some of which are visionary?
3. Does the acceptance of VR differ between genders?
4. Are there correlations between the acceptance of VR and the particular student’s semester, age, computer affinity, and prior VR experience?

## 2. Method

In order to answer these questions, medical students from German-speaking countries were asked to participate voluntarily and without remuneration in the online study (see table 1 (Tab. 1)). The majority of the students were enrolled at the University of Ulm and were invited twice via e-mail to participate in the study. Of the students that were contacted, 7.7% responded and participated in the study. At the remaining universities, students were made aware of the study via campus websites and student councils on social media. The anonymous data collection took approximately 15 to 20 minutes for each student and was conducted from January 02, 2020 to July 31, 2020. Upon request, the ethics committee of the University of Ulm decided that the study project did not present a problem and did not require an ethics vote.

**Figure 1:**
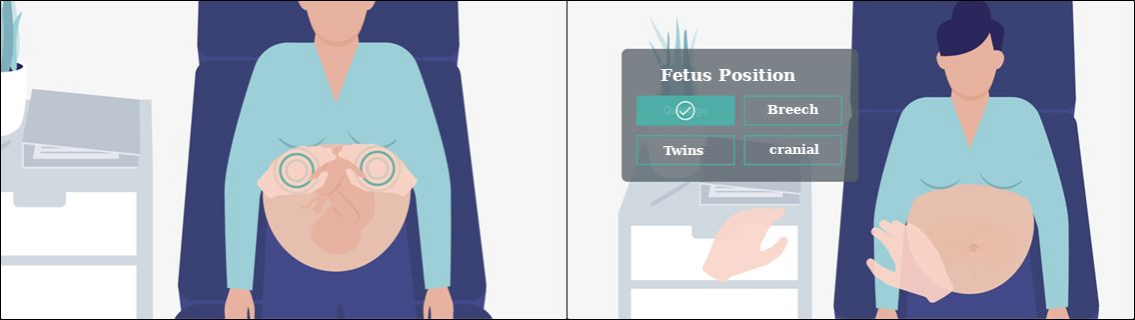
Video screenshot of the haptics function in the teaching (left) and OSCE scenario (right)

An invitation link directed students to the online study where they were asked to watch ten short videos. The videos, which can be accessed on YouTube, demonstrate and explain five levels of functionality of VR technology from the user's perspective (see figure 1 (Fig. 1)) in two different application scenarios (teaching and the OSCE) (see table 2 (Tab. 2)). The level of functionality shown increases successively and cumulatively from market-ready (visual and audiovisual stimulation) to visionary functions (haptics, oral communication, and adaptive feedback/autonomous testing) in both scenarios. Obstetrics was chosen as the application example because students are rarely able to observe and practice in real delivery rooms due to the intimacy surrounding pregnancy and childbirth.

After each video, students were asked to rate the demonstrated use of VR on the basis of acceptance indicators (e.g., presumed learning curve and innovativeness) (see table 3 (Tab. 3)). To do so, students answered items on 6-point Likert-type scales (*“strongly disagree”* to *“strongly agree”*). Since the items correlated with each other in a highly significant manner (see attachment 1 ), they were also aggregated and averaged to obtain an overall acceptance score for each video.

In order to measure the acceptance indicators individually and in an aggregated manner between (1) the application scenarios and (2) the levels of functionality, Wilcoxon signed-rank tests as well as Friedman tests followed by Dunn-Bonferroni post-hoc tests were performed. Mean value comparisons between (3) the genders were addressed with Mann-Whitney U tests, and finally (4) the correlations between the overarching acceptance and the variables semester, age, computer affinity *(“You love to work with computers”*) and prior VR (*“You have already had some form of experience with VR experiences”*) were analyzed using Spearman correlations.

## 3. Results

### 3.1. Acceptance of the level of functionality regarding teaching and the OSCE

The use of VR depicted in the videos was significantly more accepted in teaching than in the OSCE across all levels of functionality (see table 4 (Tab. 4)).

#### 3.2. Acceptance differences between the levels of functionality

**Figure 2:**
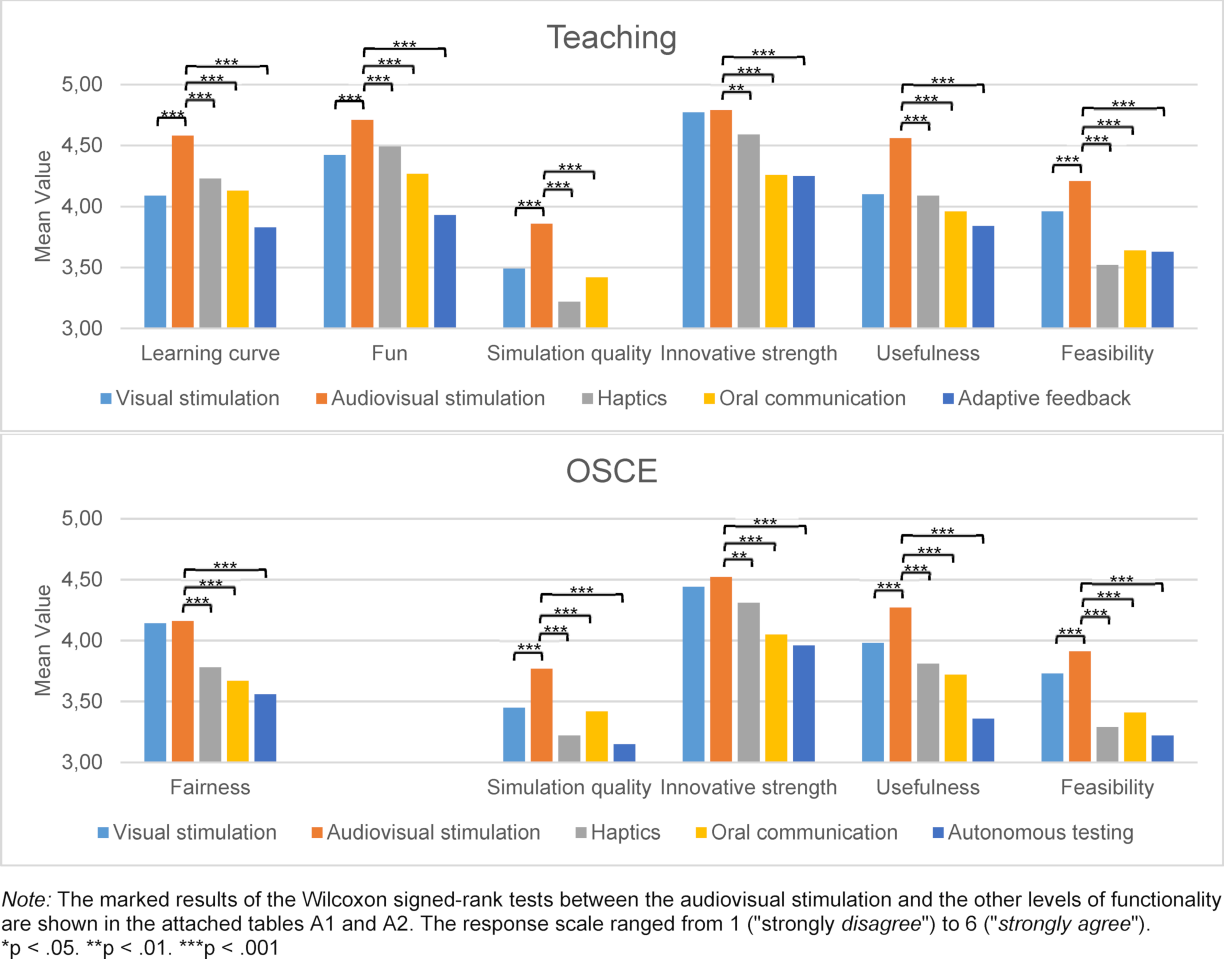
Acceptance indicators by level of functionality and application scenario

In both teaching (*Χ^2^*(4)=97.41, *p*<.001, n=318) and the OSCE (*Χ^2^*(4)=138.07, *p*<.001, n=318), VR acceptance differed significantly between the levels of functionality. The post-hoc testing (see table 5 (Tab. 5)) specified that the audiovisual degree of functionality was significantly best accepted in both application scenarios. Visual stimulation was the second preference but was only rated significantly better than the more complex haptic, oral communication and autonomous levels of functionality in the OSCE. Students rated the latter the lowest in the OSCE with a significant margin. A differentiated look at the individual acceptance indicators shows that all items scored significantly highest for the audiovisual stimulation (see figure 2 (Fig. 2), see attachment 1 , table A1 and table A2). The exception is the visual stimulation, which was only descriptively rated lower for the innovativeness and fairness indicators.

#### 3.3. Acceptance differences between the sexes

Male students exhibited a higher overarching acceptance than female students in all levels of functionality and application scenarios. Regarding the OSCE scenario videos, this gender difference became significant with regard to the functional levels of audiovisual stimulation (*U*=10048.00, *Z*=-2.00, *p*=.045, *d_Cohen_*=.23) and autonomous examination (*U*=9950.50, *Z*=-2.12, *p*=. 034, *d_Cohen_*=.24). In addition, the computer affinity was also higher among male students (*U*=7206.50, *Z*=-5.79, *p*<.000, *d_Cohen_*=. 67).

#### 3.4. Relationship between overarching acceptance and control factors

The acceptance scores across the application scenarios correlated strongly between the levels of functionality (*r*=.493 to .851) and moderately with regard to the individual computer affinity (**r**=.229 to .372) (see attachment 1 , table A3). In contrast, the control factors of age, semester and prior VR experience did not show a significant relationship with student acceptance.

## 4. Discussion

The high didactic potential of VR contrasts with the point of view of students that the technology only has a relatively low significance for current and future teaching. This discrepancy was examined in a differentiated manner in the present study in order to gear the further technical development and the implementation of VR into the curriculum towards the target group. With this goal “in mind”, the student acceptance between different (1) application scenarios and (2) levels of functionality was compared. In addition, it was checked whether, with regard to the acceptance, (3) gender differences or (4) correlations between the factors of semester, age, computer affinity and previous VR experience exist.

As in previous surveys [19], [27], the acceptance of the use of VR was generally moderate.

Referencing the comparisons made, however, this general statement is not specific enough. Across all levels of functionality, for example, VR was found to be more accepted in teaching than in the OSCE. This suggests that students have doubts about whether the novel VR technology can reliably support practical performance assessments or even automate them in the future. With regard to the Technology Acceptance Model [28], the low acceptance of autonomous VR examinations can probably be attributed to the lack of control over the system. Comparisons between levels of functionality reveal further reservations about the technology. The market-ready status quo of VR (visual and audiovisual stimulation) was significantly better accepted than the features (haptics, oral communication and adaptive feedback/autonomous testing) that are still under development in both application scenarios. Medical students thus appear to be skeptical about technological innovations that have not yet been proven in practice. This skepticism is most pronounced for autonomous VR examinations and among female students.

Since there are only few empirical arguments for the visionary VR features, this reluctance is especially understandable when it comes to examination scenarios that are directly relevant to a student’s grading. In detail, however, the results are counterintuitive. As the level of functionality increased, not only did the assumed feasibility decrease but so did the innovativeness, simulation quality, meaningfulness, and learning curve. The same is true for the application-specific factors of fun, learning curve, and fairness. These statements contradict the potential attributed to the technical innovations. While haptic feedback is primarily intended to improve the immersion and quality of the simulation, the integration of artificial intelligence in VR promises individual learning and examination experiences with lifelike virtual patients. These potentials are not reflected in the assessment of the medical students. One reason for this counterintuitive assessment may be the human tendency to embrace the ordinary (audiovisual stimulation) and to avoid the unknown (e.g., autonomous performance assessment) [29]. This basic attitude is substantially reflected in the positive correlation between acceptance and computer affinity. Since the use of haptic feedback and AI is still in development and thus generally unknown, student skepticism towards visionary levels of functionality can possibly be attributed to a lack of experience.

The restrained acceptance of the market-ready status quo can possibly be explained in a similar manner. In a cross-curriculum survey by Weisflog and Böckel [19], students considered VR more important for their studies if the technology was available to them at their university. In 2019, however, only about 4% of the students in Germany had VR equipment at their disposal on campus. Even in the private sector, the use of VR – despite increasing hardware and software sales – is currently still limited to a technology-savvy minority [https://www2.deloitte.com/de/de/pages/presse/contents/zukunftsperspektiven-fuer-virtual-augmented-reality.html]. Consequently, only a small percentage of students has had the opportunity to make practical use of the current VR technology and overcome possible reservations. However, this explanatory approach is not supported by the prior VR experience measured in the study. The corresponding item presumably fails to express the individual level of knowledge of VR technology since the quality and quantity of the VR experiences were not surveyed.

Also noteworthy is the consistently high correlation between the acceptance indicators (see attachment 1 , table A3). The high internal consistency suggests that many medical students have an established opinion about the use of VR that has conditioned their responses across application scenarios and levels of functionality. The transferability of these findings needs to be critically discussed, however. The low response rate of 7.7% in Ulm limits the representativeness of the sample. In addition, the video demonstrations referred exclusively to obstetrics, which means that the acceptance values can be concluded for other fields of application only indirectly. Finally, when interpreting the results, it must also be taken into account that the students only evaluated videos on the use of VR since the higher levels of functionality have not yet reached market maturity. In line with the assumption that the acceptance of VR increases as awareness rises, the assessment would probably be more positive if students had had a practical demonstration.

Regardless, however, the study results make it clear that the concerns and technical knowledge of students must be taken into account in the further development and the implementation of VR in the curriculum. In order for real learning effects to result from the potential of VR, the technology should be introduced not only as a teaching tool but also be part of the medical studies curriculum. The opportunity to learn about the application of VR in a guided manner and to discuss it critically also promotes the media competence of medical students who will possibly work with VR in their future daily clinical routine (e.g., simulation-based planning of surgical interventions). Whether VR will also be suitable for conducting practical examinations or whether student skepticism will continue to be justified in the future should be scientifically examined as soon as the technology has become established in teaching and proven to be reliable.

## 5. Conclusion

The differentiated assessment of the acceptance of VR by students revealed that the subjectively low perceived importance of VR is due to a skepticism towards emerging technologies. Medical students seem to have too little knowledge about VR to adequately assess its didactic potential. In order for medical students to become familiar with the technology and to be able to use it competently in their everyday clinical practice in the future, VR should be introduced not only as a teaching tool but also integrated into the curriculum. The implementation of practical examinations in VR, on the other hand, is only recommended once the technology has proven itself to be reliable in teaching.

## Authors

The authors S. Walter and R. Speidel share the first authorship.

## Competing interests

The authors declare that they have no competing interests. 

## Supplementary Material

Attachment 1

## Figures and Tables

**Table 1:**
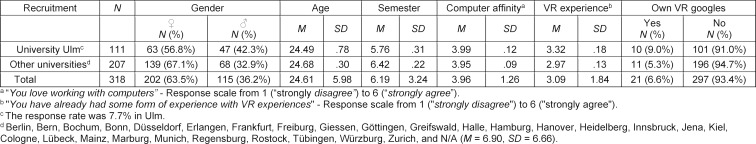
Sample description

**Table 2:**
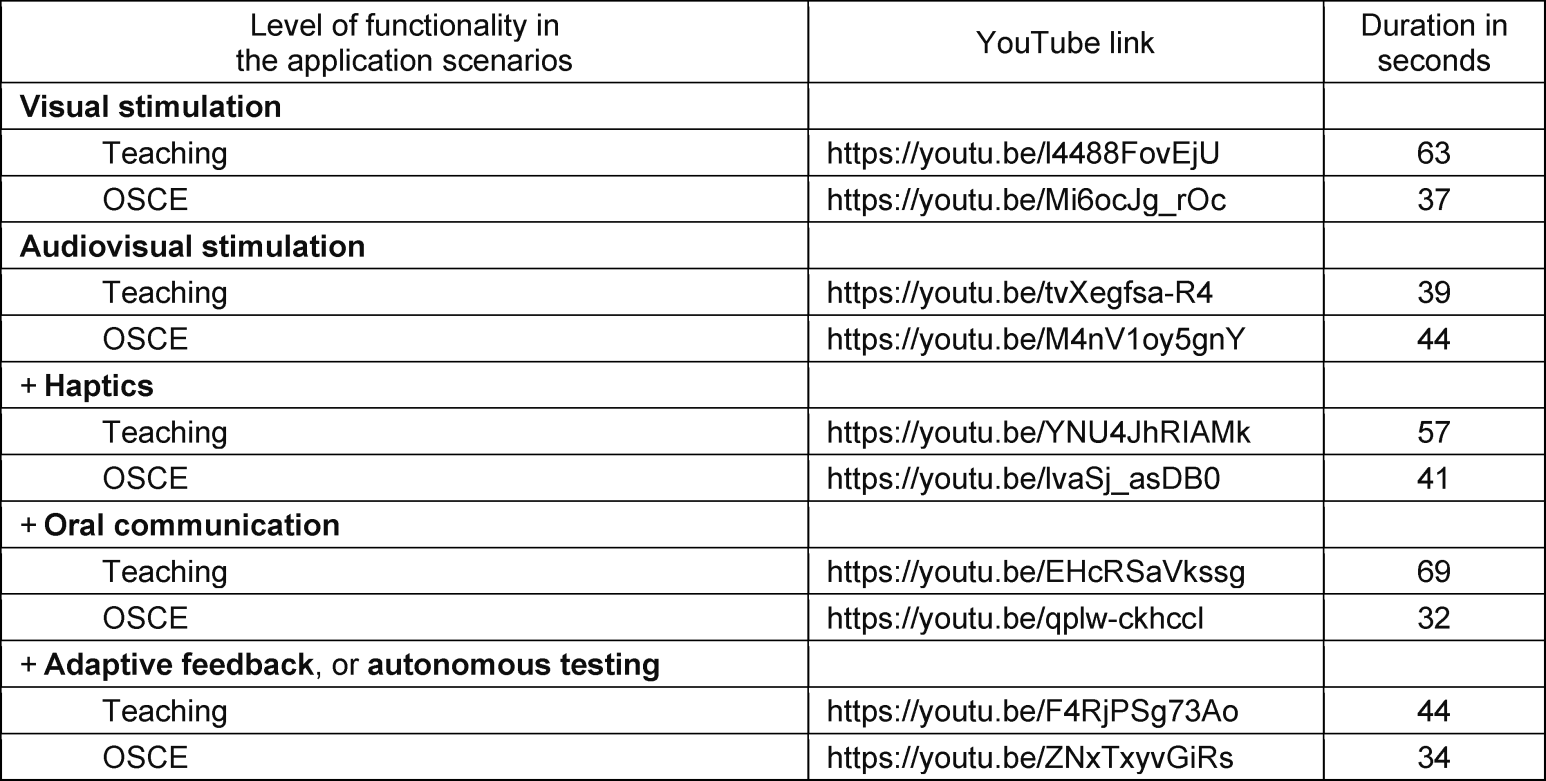
YouTube links and duration of the videos

**Table 3:**
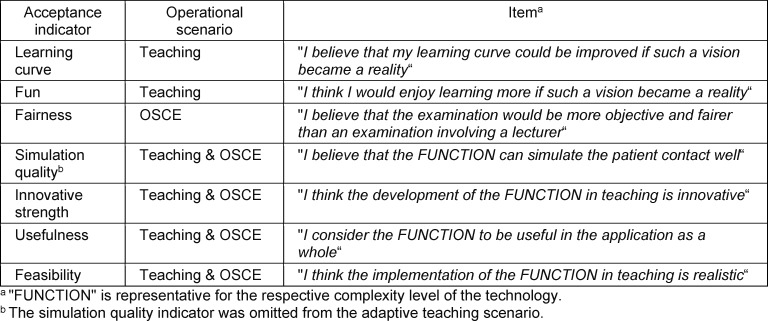
Acceptance indicators

**Table 4:**
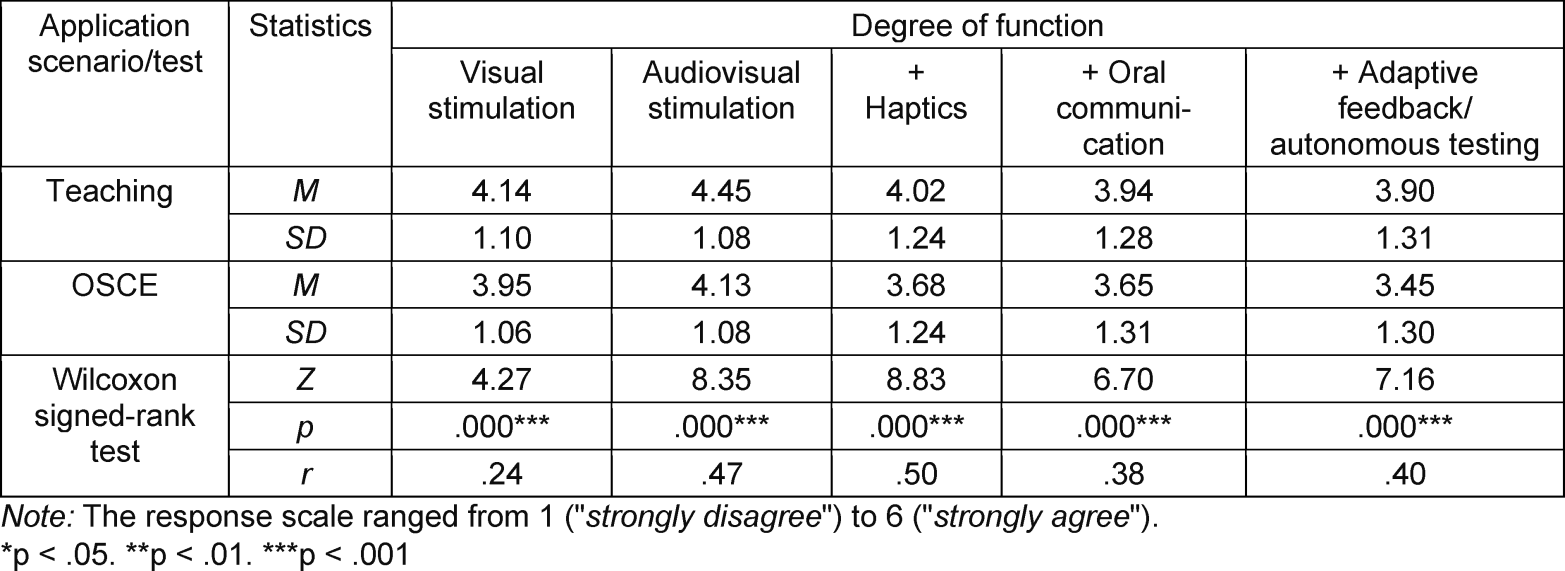
Overarching acceptance of levels of functionality between teaching and the OSCE

**Table 5:**
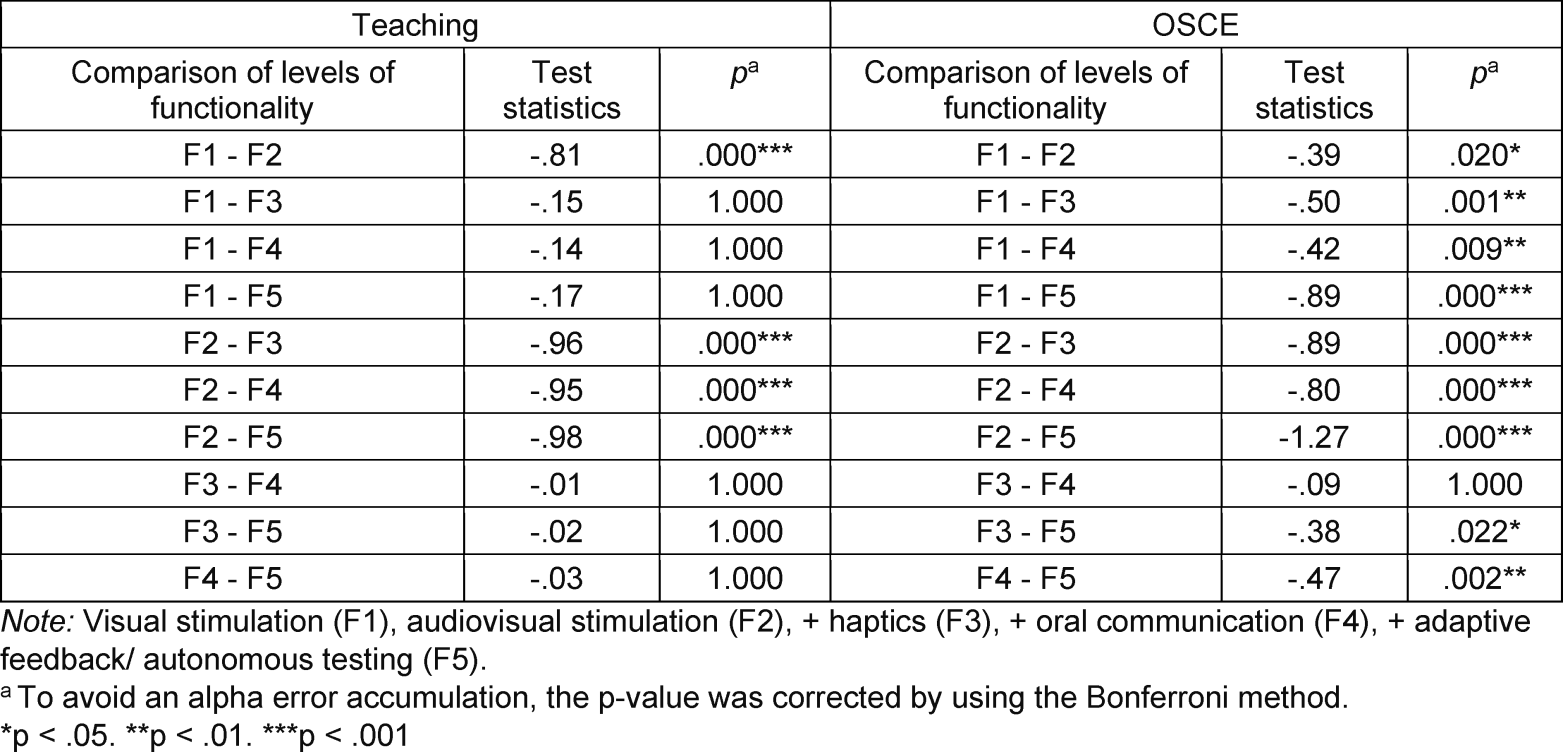
Dunn-Bonferroni post-hoc tests on the overall acceptance between levels of functionality

## References

[1] Boshuizen H, Schmidt H (2008). The development of clinical reasoning expertise. Clin Reason Health Prof.

[2] Keifenheim KE, Teufel M, Ip, J, Speiser N, Leehr EJ, Zipfel S, Herrmann-Werner A (2015). Teaching history taking to medical students: A systematic review. BMC Med Educ.

[3] Kiesewetter J, Kager M, Lux R, Zwissler B, Fischer MR, Dietz I (2014). German undergraduate medical students' attitudes and needs regarding medical errors and patient safety - a national survey in Germany. Med Teach.

[4] Cook DA (2014). How much evidence does it take? A cumulative meta-analysis of outcomes of simulation-based education. Med Educ.

[5] Kononowicz AA, Woodham LA, Edelbring S, Stathakarou N, Davies D, Saxena N, Tudor Car L, Carlsted-Duke J, Car J, Zary N (2019). Virtual patient simulations in health professions education: Systematic review and meta-analysis by the digital health education collaboration. J Med Internet Res.

[6] Huwendiek S, De leng BA, Zary N, Fischer MR, Ruiz JG, Ellaway R (2009). Towards a typology of virtual patients. Med Teach.

[7] Bokken L, Rethans JJ, Scherpbier AJ, van der Vleuten CP (2008). Strengths and weaknesses of simulated and real patients in the teaching of skills to medical students: a review. Simul Healthc.

[8] Cook DA, Hamstra SJ, Brydges R, Zendejas B, Szostek JH, Wang AT, Erwin PJ, Hatala R (2013). Comparative effectiveness of instructional design features in simulation-based education: Systematic review and meta-analysis. Med Teach.

[9] Shorey S, Ang E, Yap J, Ng ED, Lau ST, Chui CK (2019). A Virtual Counseling Application Using Artificial Intelligence for Communication Skills Training in Nursing Education: Development Study. J Med Internet Res.

[10] Talbot TB, Rizzo AS, Dubbels BR (2019). Virtual standardized patients for interactive conversational training: A grand experiment and new approach.

[11] Slater M, Wilbur S (1997). A Framework for Immersive Virtual Environments (FIVE): Speculations on the Role of Presence in Virtual Environments. Presence.

[12] Chirico A, Gaggioli A (2019). When Virtual Feels Real: Comparing Emotional Responses and Presence in Virtual and Natural Environments. Cyberpsychol Behav Soc Netw.

[13] Kolb DA (1984). Experiential learning: Experience as the source of learning and development.

[14] Kwon C (2019). Verification of the possibility and effectiveness of experiential learning using HMD-based immersive VR technologies. Virt Reality.

[15] Chavez B, Bayona S, Rocha Á, Adeli H, Reis LP, Costanzo S (2018). Virtual Reality in the Learning Process. Advances in Intelligent Systems and Computing. Trends and Advances in Information Systems and Technologies.

[16] Kyaw BM, Saxena N, Posadzki P, Vseteckova J, Nikolaou CK, George PP, Divakar U, Masiello I, Kononowicz AA, Zary N, Tudor Car L (2019). Virtual Reality for Health Professions Education: Systematic Review and Meta-Analysis by the Digital Health Education Collaboration. J Med Internet Res.

[17] Pourmand A, Davis S, Lee D, Barber S, Sikka N (2017). Emerging Utility of Virtual Reality as a Multidisciplinary Tool in Clinical Medicine. Games Health J.

[18] Cipresso P, Giglioli IAC, Raya MA, Riva G (2018). The Past, Present, and Future of Virtual and Augmented Reality Research: A Network and Cluster Analysis of the Literature. Front Psychol.

[19] Weisflog W, Böckel A (2020). Ein studentischer Blick auf den Digital Turn: Auswertung einer bundesweiten Befragung von Studierenden für Studierende. Arbeitspapier Nr. 54.

[20] Kuhn S, Frankenhauser S, Tolks D (2018). Digitale Lehr- und Lernangebote in der medizinischen Ausbildung: Schon am Ziel oder noch am Anfang?. Bundesgesundheitsblatt Gesundheitsforschung Gesundheitsschutz.

[21] Pottle J (2019). Virtual reality and the transformation of medical education. Future Healthc J.

[22] Zender R, Weise M, von der Heyde M, Söbke H, Krömker D, Schroeder U (2018). Lehren und Lernen mit VR und AR - Was wird erwartet? Was funktioniert?. DeLFI 2018 - Die 16. E-Learning Fachtagung Informatik.

[23] Frithioff A, Frendø M, Mikkelsen PT, Sørensen MS, Andersen SA (2020). Ultra-high-fidelity virtual reality mastoidectomy simulation training: a randomized, controlled trial. Eur Arch Otorhinolaryngol.

[24] Rangarajan K, Davis H, Pucher PH (2020). Systematic Review of Virtual Haptics in Surgical Simulation: A Valid Educational Tool?. J Surg Educ.

[25] Mirchi N, Bissonnette V, Yilmaz R, Ledwos N, Winkler-Schwartz A, Del Maestro RF (2020). The Virtual Operative Assistant: An explainable artificial intelligence tool for simulation-based training in surgery and medicine. PLoS ONE.

[26] Veneziano D, Cacciamani G, Rivas JG, Marino NS, Bhaskar K (2020). VR and machine learning: novel pathways in surgical hands-on training. Curr Opin Urol.

[27] Speidel R, Schneider A, Koerner J, Grab-Kroll C, Oechsner W (2021). Did video kill the XR star? Digital trends in medical education before and after the COVID- 19 outbreak from the perspective of students and lecturers from the faculty of medicine at the University of Ulm. GMS J Med Educ.

[28] Holden RJ, Karsh BT (2010). The technology acceptance model: its past and its future in health care. J Biomed Inform.

[29] Castro D, McQuinn A (2015). The Privacy Panic Cycle: A Guide to Public Fears About New Technologies.

