# Supplementary material for: Skepticism towards advancing VR technology – student acceptance of VR as a teaching and assessment tool in medicine
Source: GMS J Med Educ. 2021 Sep 15;38(6):Doc100. doi: 10.3205/zma001496 (PMC8493843; doi:10.3205/zma001496)
Supplement: Attachment 1 [file JME-38-6-100-s-001.pdf]

Attachment 1:

Table A1

Comparison of acceptance indicators in the teaching scenario with Wilcoxon signed-rank tests

Wilcoxon signed-rank test	*Learning curve*				*Fun*				*Simulation quality*				*Innovative strength*				*Usefulness*				*Feasibility*			
	F2 – F1	*F2 – F3*	*F2 – F4*	*F2 – F5*	*F2 – F1*	*F2 – F3*	*F2 – F4*	*F2 – F5*	*F2 – F1*	*F2 – F3*	*F2 – F4*	*F2 – F5*	*F2 – F1*	*F2 – F3*	*F2 – F4*	*F2 – F5*	*F2 – F1*	*F2 – F3*	*F2 – F4*	*F2 – F5*	*F2 – F1*	*F2 – F3*	*F2 – F4*	*F2 – F5*
Z	-8.39	-5.32	-6.96	-9.46	-5.68	-4.02	-6.60	-9.23	-5.98	-7.83	-6.01		-.15	-3.07	-7.31	-6.89	-4.92	-9.05	-7.47	-7.90	-7.24	-6.15	-7.42	-8.36
p	.000***	.000***	.000***	.000***	.000***	.000***	.000***	.000***	.000***	.000***	.000***		.885	.002**	.000***	.000***	.000***	.000***	.000***	.000***	.000***	.000***	.000***	.000***

Note. Visual stimulation (F1), audiovisual stimulation (F2), + haptics (F3), + oral communication (F4), + adaptive feedback/ autonomous testing (F5)
*p < .05. **p < .01. ***p < .001

Table A2

Comparison of acceptance indicators in the OSCE scenario with Wilcoxon signed-rank tests

Wilcoxon signed-rank test	*Fairness*				*Simulation quality*				*Innovative strength*				*Usefulness*				*Feasibitliy*			
	F2 – F1	*F2 – F3*	*F2 – F4*	*F2 – F5*	*F2 – F1*	*F2 – F3*	*F2 – F4*	*F2 – F5*	*F2 – F1*	*F2 – F3*	*F2 – F4*	*F2 – F5*	*F2 – F1*	*F2 – F3*	*F2 – F4*	*F2 – F5*	*F2 – F1*	*F2 – F3*	*F2 – F4*	*F2 – F5*
Z	-.45	-5.41	-6.82	-7.35	-5.94	-7.17	-4.80	-8.46	-1.78	-3.17	-6.68	-7.44	-5.76	-5.92	-6.47	-9.62	-3.75	-7.99	-6.42	-8.80
p	.651	.000***	.000***	.000***	.000***	.000***	.000***	.000***	.076	.002**	.000***	.000***	.000***	.000***	.000***	.000***	.000***	.000***	.000***	.000***

Note. Visual stimulation (F1), audiovisual stimulation (F2), + haptics (F3), + oral communication (F4), + adaptive feedback/ autonomous testing (F5)
*p < .05. **p < .01. ***p < .001

Attachment 1 to: Walter S, Speidel RA, Hann A, Leitner J, Jerg-Bretzke L, Kropp P, Sheaf J, Ebner F. *Skepticism towards advancing VR technology – Student acceptance of VR as a teaching and assessment tool in medicine*. GMS J Med Educ. 2021;38(6):Doc100. DOI: 10.3205/zma001496

Table A3

Correlation between overarching acceptance, computer affinity and VR experience

Level of functionality and control factors	Teaching					OSCE					Computer affinity
	F1	F2	F3	F4	F5	F1	F2	F3	F4	F5	
Teaching											
F1	-	.774**	.605**	.643**	.618**	.726**	.707**	.578**	.618**	.540**	.335**
F2	.774**	-	.603**	.639**	.605**	.657**	.772**	.524**	.559**	.507**	.369**
F3	.605**	.603**	-	.674**	.515**	.493**	.669**	.851**	.610**	.512**	.252**
F4	.643**	.639**	.674**	-	.587**	.517**	.646**	.662**	.829**	.594**	.275**
F5	.618**	.605**	.515**	.587**	-	.604**	.607**	.520**	.589**	.646**	.293**
OSCE											
F1	.726**	.657**	.493**	.517**	.604**	-	.822**	.596**	.609**	.634**	.291**
F2	.707**	.772**	.669**	.646**	.607**	.822**	-	.725**	.664**	.639**	.372**
F3	.578**	.524**	.851**	.662**	.520**	.596**	.725**	-	.698**	.639**	.229**
F4	.618**	.559**	.610**	.829**	.589**	.609**	.664**	.698**	-	.702**	.313**
F5	.540**	.507**	.512**	.594**	.646**	.634**	.639**	.639**	.702**	-	.239**
Computer affinity	.335**	.369**	.252**	.275**	.293**	.291**	.370**	.229**	.313**	.239**	-

Note. Visual stimulation (F1), audiovisual stimulation (F2), + haptics (F3), + oral communication (F4), + adaptive feedback/ autonomous testing (F5)
*p < .05. **p < .01. ***p < .001
